# Supplementary material for: Carbonyl Composition and Electrophilicity in Vaping Emissions of Flavored and Unflavored E-Liquids
Source: Toxics. 2021 Dec 9;9(12):345. doi: 10.3390/toxics9120345 (PMC8705255; doi:10.3390/toxics9120345)
Supplement: Supplementary file 1 [file toxics-09-00345-s001.zip › toxics-1458595-supplementary.pdf]

toxics MDPI

Supplementary Materials: Carbonyl Composition and Electrophilicity in Vaping Emissions of Flavored and Unflavored E-Liquids

Jin Y. Chen, Alexa Canchola, and Ying-Hsuan Lin

Table S1. Optimized geometries of target carbonyls calculated by DFT/B3LYP/6-311+G(d, p) level of theory and in water solvation using Gaussian 16W program.

Formaldehyde

Charge = 0
E(UB3LYP) = −114.432708094 Hartree (Eh)
Electronic state: 1-A
Cartesian Coordinates (Angstroms):

O	−0.000041	0.676752	0.000000
C	−0.000041	−0.531376	0.000000
H	−0.938433	−1.112848	0.000000
H	0.939008	−1.112912	0.000000

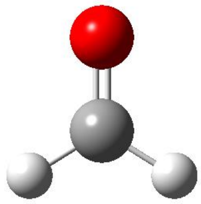

Charge = −1
E(UB3LYP) = −114.624580950 Hartree (Eh)
Electronic state: 1-A
Cartesian Coordinates (Angstroms):

O	−0.000009	0.727861	0.000000
C	−0.000009	−0.584091	0.000000
H	−0.936931	−1.159157	0.000000
H	0.937051	−1.159186	0.000000

Charge = +1
E(UB3LYP) = −114.160019523 Hartree (Eh)
Electronic state: 1-A
Cartesian Coordinates (Angstroms):

O	−0.000105	0.663897	0.000000
C	−0.000105	−0.522196	0.000000
H	−0.962402	−1.089947	0.000000
H	0.963872	−1.088057	0.000000

Acetaldehyde

Charge = 0
E(UB3LYP) = −153.354522985 Hartree (Eh)
Electronic state: 1-A
Cartesian Coordinates (Angstroms):

C	0.000000	0.471652	0.000000
O	−1.207923	0.355997	0.000000
C	0.949642	−0.693883	0.000000
H	0.452229	1.482132	0.000000
H	1.989744	−0.367922	0.000000

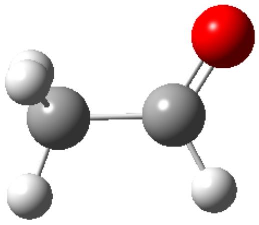

H	0.761779	−1.314399	0.881325
H	0.761779	−1.314399	−0.881325

Charge = −1

E(UB3LYP) = −153.370677836 Hartree (Eh)

Electronic state: 1-A

Cartesian Coordinates (Angstroms):

C	0.000000	0.497419	0.000000
O	−1.293321	0.239851	0.000000
C	1.016429	−0.611764	0.000000
H	0.360356	1.536615	0.000000
H	2.038719	−0.214048	0.000000
H	0.924458	−1.277656	0.878966
H	0.924458	−1.277656	−0.878966

Charge = +1

E(UB3LYP) = −153.102042126 Hartree (Eh)

Electronic state: 1-A

Cartesian Coordinates (Angstroms):

C	0.000000	0.451208	0.000000
O	−1.192126	0.398092	0.000000
C	0.949359	−0.730416	0.000000
H	0.434009	1.484123	0.000000
H	1.968672	−0.358366	0.000000
H	0.719088	−1.317621	0.893620
H	0.719088	−1.317621	−0.893620

Acrolein

Charge = 0

E(UB3LYP) = −191.183387804 Hartree (Eh)

Electronic state: 1-A

Cartesian Coordinates (Angstroms):

C	−1.214748	1.279427	0.000000
C	0.000000	0.719262	0.000000
C	0.148409	−0.739513	0.000000
O	1.219144	−1.322395	0.000000
H	−2.112691	0.668793	0.000000
H	−1.351926	2.354067	0.000000
H	0.910131	1.311027	0.000000
H	−0.800630	−1.309782	0.000000

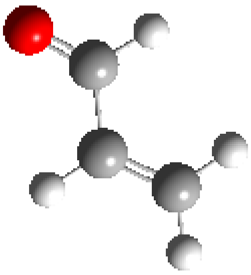

Charge = −1

E(UB3LYP) = −191.208242254 Hartree (Eh)

Electronic state: 1-A

Cartesian Coordinates (Angstroms):

C	−1.186351	1.404274	0.000000
C	0.000000	0.667011	0.000000
C	0.114150	−0.735584	0.000000
O	1.208609	−1.417489	0.000000
H	−2.156618	0.915291	0.000000

H	−1.178328	2.487565	0.000000
H	0.944178	1.215554	0.000000
H	−0.844904	−1.292697	0.000000

Charge = +1

E(UB3LYP) = −190.930698073 Hartree (Eh)

Electronic state: 1-A

Cartesian Coordinates (Angstroms):

C	−0.923399	1.490212	0.000000
C	0.172970	0.709976	0.000000
C	0.000000	−0.719132	0.000000
O	0.885347	−1.559127	0.000000
H	−1.930249	1.090204	0.000000
H	−0.813474	2.568501	0.000000
H	1.188085	1.083768	0.000000
H	−1.024564	−1.155795	0.000000

Benzaldehyde

Charge = 0

E(UB3LYP) = −345.676360401 Hartree (Eh)

Electronic state: 1-A

Cartesian Coordinates (Angstroms):

O	2.854181	−0.391611	−0.000078
C	1.986641	0.463916	0.000076
C	0.535584	0.200982	0.000040
C	0.034053	−1.109905	0.000036
C	−0.349770	1.286844	0.000010
C	−1.337636	−1.325852	0.000002
C	−1.725199	1.067938	−0.000021
C	−2.217196	−0.237321	−0.000027
H	2.262757	1.535775	−0.000056
H	0.728135	−1.941920	0.000060
H	0.042746	2.298463	0.000017
H	−1.728700	−2.336551	0.000000
H	−2.409849	1.907725	−0.000044
H	−3.287394	−0.410225	−0.000052

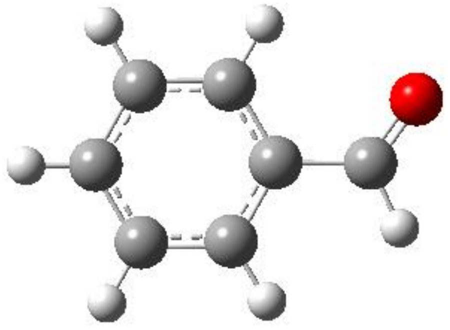

Charge = −1

E(UB3LYP) = −345.763742749 Hartree (Eh)

Electronic state: 1-A

Cartesian Coordinates (Angstroms):

O	2.905663	−0.399770	−0.000288
C	1.972124	0.479781	0.000285
C	0.571895	0.221027	0.000170
C	0.032434	−1.108894	0.000139
C	−0.377215	1.294520	0.000046
C	−1.336634	−1.327406	0.000006
C	−1.739152	1.059533	−0.000084
C	−2.249060	−0.257790	−0.000107
H	2.251592	1.550684	−0.000317
H	0.718860	−1.948297	0.000218

H	−0.007260	2.317049	0.000055
H	−1.710758	−2.347931	−0.000009
H	−2.426349	1.900977	−0.000163
H	−3.317749	−0.438952	−0.000207

Charge = +1

E(UB3LYP) = −345.403731647 Hartree (Eh)

Electronic state: 1-A

Cartesian Coordinates (Angstroms):

O	2.820665	−0.456620	−0.000006
C	2.032758	0.460995	0.000007
C	0.555395	0.250624	0.000003
C	0.029491	−1.077953	0.000004
C	−0.313875	1.310038	0.000002
C	−1.375061	−1.320978	−0.000001
C	−1.725245	1.063451	−0.000007
C	−2.243289	−0.258955	0.000003
H	2.360204	1.513283	−0.000007
H	0.725059	−1.909611	−0.000001
H	0.044530	2.332454	0.000002
H	−1.735073	−2.341198	−0.000013
H	−2.406862	1.905372	−0.000010
H	−3.314216	−0.410675	0.000011

trans-2-Hexenal

Charge = 0

E(UB3LYP) = −309.958896545 Hartree (Eh)

Electronic state: 1-A

Cartesian Coordinates (Angstroms):

C	−2.638669	0.319521	−0.003206
C	−1.357376	−0.374257	0.034723
C	−0.244343	0.262892	0.436141
C	1.123625	−0.329315	0.513109
C	2.148214	0.426447	−0.358354
C	3.559730	−0.152190	−0.233532
O	−3.695828	−0.186820	−0.351412
H	−2.609234	1.380353	0.313763
H	−1.342381	−1.416907	−0.270720
H	−0.330353	1.310276	0.726660
H	1.459484	−0.282706	1.557916
H	1.095248	−1.385688	0.229712
H	1.823340	0.389462	−1.403455
H	2.154769	1.483431	−0.070627
H	4.264537	0.396518	−0.863782
H	3.919126	−0.096430	0.798560
H	3.584988	−1.202335	−0.540017

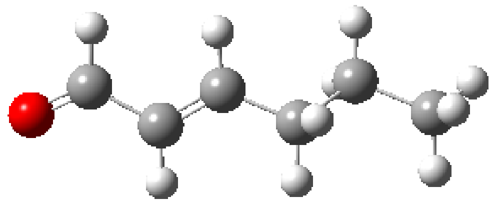

Charge = −1

E(UB3LYP) = −310.031522249 Hartree (Eh)

Electronic state: 1-A

Cartesian Coordinates (Angstroms):

C	−2.672951	0.253138	−0.140291
C	−1.376611	−0.190528	0.179681
C	−0.218484	0.587608	0.257114
C	1.141394	0.052921	0.604013
C	2.185152	0.156002	−0.529532
C	3.574874	−0.343986	−0.121547
O	−3.744389	−0.471509	−0.209510
H	−2.768165	1.337295	−0.353498
H	−1.280673	−1.259051	0.391294
H	−0.288957	1.655873	0.049252
H	1.558396	0.580524	1.478330
H	1.054235	−0.999730	0.904042
H	1.825427	−0.412510	−1.395260
H	2.252955	1.201015	−0.855850
H	4.290784	−0.258109	−0.944386
H	3.968415	0.231434	0.723064
H	3.542453	−1.395604	0.182461

Charge = +1

E(UB3LYP) = −309.687393075 Hartree (Eh)

Electronic state: 1-A

Cartesian Coordinates (Angstroms):

C	−2.585359	0.294347	0.004884
C	−1.349933	−0.406959	0.009250
C	−0.239085	0.250077	0.441860
C	1.106798	−0.350496	0.499560
C	2.133001	0.453198	−0.344018
C	3.536416	−0.145962	−0.233474
O	−3.682338	−0.140600	−0.344444
H	−2.622612	1.354055	0.336706
H	−1.347230	−1.434081	−0.332454
H	−0.330998	1.285572	0.762097
H	1.426822	−0.312376	1.550862
H	1.086097	−1.397333	0.190107
H	1.808354	0.456420	−1.388058
H	2.139440	1.493539	−0.006288
H	4.241349	0.428485	−0.839553
H	3.891547	−0.134177	0.800658
H	3.554909	−1.180531	−0.586898

Table S2. Condensed Fukui parameters (f_k^0, f_k^-, f_k^+, and dual-descriptor) calculated using NPA data by UCA-Fukui software.

Atom	NPA neutral	NPA anion	NPA cation	f_k^-	f_k^+	f_k^0	Dual descriptor
				Formaldehyde			
O	−0.5521	−0.8986	0.0149	0.5670	0.3465	0.4568	−0.2205
C	0.3153	−0.2770	0.3305	0.0152	0.5923	0.3037	0.5771
H	0.1184	0.0878	0.3272	0.2088	0.0306	0.1197	−0.1782
H	0.1184	0.0878	0.3274	0.2090	0.0306	0.1198	−0.1785
				Acetaldehyde			
C	0.4636	−0.0180	0.4809	0.0172	0.4817	0.2494	0.4644
O	−0.5898	−0.9263	−0.0454	0.5445	0.3365	0.4405	−0.2080
C	−0.6949	−0.6606	−0.6248	0.0702	−0.0343	0.0179	−0.0358
H	0.1196	0.0842	0.3307	0.2110	0.0354	0.1232	−0.1756
H	0.2206	0.1893	0.2707	0.0501	0.0313	0.0407	−0.0188
H	0.2405	0.1657	0.2940	0.0535	0.0747	0.0641	0.0212
H	0.2405	0.1657	0.2940	0.0535	0.0747	0.0641	0.0212
				Acrolein			
C	−0.2567	−0.5583	−0.0798	0.1769	0.3016	0.2392	0.1246
C	−0.3109	−0.3792	−0.3171	−0.0063	0.0684	0.031	0.0621
C	0.4033	0.1652	0.3788	−0.0245	0.238	0.1068	0.2136
O	−0.5864	−0.8367	−0.036	0.5504	0.2503	0.4003	−0.3001
H	0.1989	0.169	0.2262	0.0272	0.0299	0.0286	0.0027
H	0.2086	0.1774	0.2371	0.0285	0.0313	0.0299	0.0028
H	0.2198	0.1766	0.2731	0.0534	0.0432	0.0483	−0.0102
H	0.1233	0.0859	0.3177	0.1944	0.0374	0.1159	−0.157
				Benzaldehyde			
O	−0.5885	−0.8144	−0.5189	0.0696	0.2260	0.1564	−0.3404
C	0.4320	0.1894	0.4330	0.0010	0.2426	0.2416	0.1945
C	−0.1804	−0.1998	−0.1452	0.0352	0.0194	−0.0157	0.0129
C	−0.1554	−0.2622	0.1126	0.2681	0.1067	−0.1614	0.0780
C	−0.1572	−0.2296	−0.0936	0.0636	0.0724	0.0088	0.0173
C	−0.2082	−0.2243	−0.1296	0.0786	0.0161	−0.0625	−0.0080
C	−0.2122	−0.2422	0.0807	0.2928	0.0300	−0.2628	0.0203
C	−0.1686	−0.3120	−0.1507	0.0179	0.1435	0.1256	0.0809
H	0.1267	0.0942	0.1546	0.0279	0.0325	0.0046	−0.1421
H	0.2266	0.2038	0.2483	0.0217	0.0229	0.0012	0.0085
H	0.2217	0.1970	0.2554	0.0337	0.0247	−0.0090	0.0042
H	0.2212	0.1993	0.2543	0.0331	0.0219	−0.0112	0.0016
H	0.2219	0.2005	0.2437	0.0218	0.0215	−0.0003	0.0024
H	0.2203	0.2004	0.2554	0.0352	0.0199	−0.0153	0.0082
				trans-2-Hexenal			
C	0.4029	0.1602	0.3721	−0.0307	0.2426	0.1059	0.2119
C	−0.3295	−0.3857	−0.3162	0.0133	0.0563	0.0348	0.0430
C	−0.0420	−0.3039	0.0912	0.1332	0.2619	0.1976	0.1287
C	−0.4341	−0.4094	−0.4456	−0.0116	−0.0247	0.0181	0.0131
C	−0.3755	−0.3794	−0.3722	0.0034	0.0039	0.0036	0.0005
C	−0.5761	−0.5803	−0.5745	0.0016	0.0042	0.0029	0.0026
O	−0.6050	−0.8196	−0.0606	0.5443	0.2146	0.3795	−0.3298
H	0.1202	0.0676	0.3094	0.1892	0.0526	0.1209	−0.1366
H	0.2142	0.1757	0.2647	0.0505	0.0385	0.0445	−0.0120
H	0.2012	0.1615	0.2244	0.0233	0.0397	0.0315	0.0164
H	0.2236	0.1781	0.2559	0.0323	0.0455	0.0389	0.0132
H	0.2087	0.1871	0.2253	0.0166	0.0216	0.0191	0.0050
H	0.1961	0.1842	0.2054	0.0093	0.0120	0.0106	0.0027
H	0.1961	0.1847	0.2049	0.0088	0.0114	0.0101	0.0026
H	0.2048	0.1964	0.2127	0.0078	0.0084	0.0081	0.0006
H	0.1971	0.1914	0.2013	0.0042	0.0057	0.0050	0.0015
H	0.1973	0.1915	0.2018	0.0044	0.0058	0.0051	0.0014

Table S3. Condensed Fukui function, f_k^-, of carbonyl compounds calculated using UCA-FUKUI software.

		f_k^- **(for electrophilic attack)**			
**compound**	**structure**	$\mathbf{O_{carbonyl}}$[1]	$\mathbf{C_{carbonyl}}$[2]	$\mathbf{C_{\alpha}}$[3]	$\mathbf{C_{\beta}}$[4]
		simple carbonyl			
formaldehyde	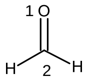	0.567	0.0152	n/a	n/a
acetaldehyde	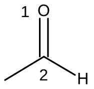	0.5445	0.0172	n/a	n/a
benzaldehyde	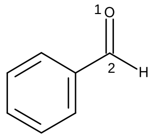	0.0696	0.0010	n/a	n/a
		α,β-unsaturated carbonyl			
acrolein	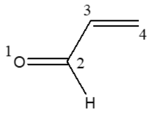	0.5504	−0.0245	−0.0063	0.1769
trans-2-hexenal	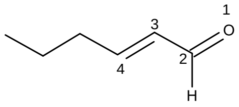	0.5443	−0.0307	0.0133	0.1332

Table S4. Condensed Fukui function, f_k^0, of carbonyl compounds calculated using UCA-FUKUI software.

		f_k^0 **(for neutral/radical attack)**			
**compound**	**structure**	$\mathbf{O_{carbonyl}}$[1]	$\mathbf{C_{carbonyl}}$[2]	$\mathbf{C_{\alpha}}$[3]	$\mathbf{C_{\beta}}$[4]
		simple carbonyl			
formaldehyde	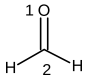	0.4568	0.3037	n/a	n/a
acetaldehyde	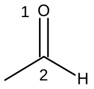	0.4405	0.2494	n/a	n/a
benzaldehyde	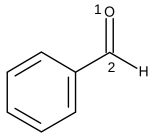	0.1564	0.2416	n/a	n/a
		α,β-unsaturated carbonyl			
acrolein	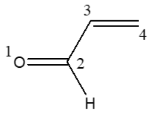	0.4003	0.1068	0.031	0.2392
trans-2-hexenal	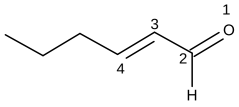	0.3795	0.1059	0.0348	0.1976

Table S5. Condensed Fukui function, dual-descriptor, of carbonyl compounds calculated using UCA-FUKUI software.

		**dual-descriptor**			
**compound**	**structure**	$\mathbf{O_{carbonyl}}^{1}$	$\mathbf{C_{carbonyl}}^{2}$	$\mathbf{C_{\alpha}}^{3}$	$\mathbf{C_{\beta}}^{4}$
simple carbonyl					
formaldehyde	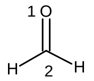	−0.2205	0.5771	n/a	n/a
acetaldehyde	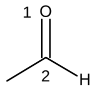	−0.2080	0.4644	n/a	n/a
benzaldehyde	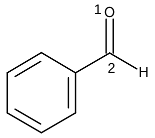	−0.3403	0.1945	n/a	n/a
α,β-unsaturated carbonyl					
acrolein	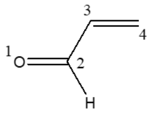	−0.3001	0.2136	0.0621	0.1246
trans-2-hexenal	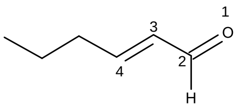	−0.3298	0.2119	0.0430	0.1287

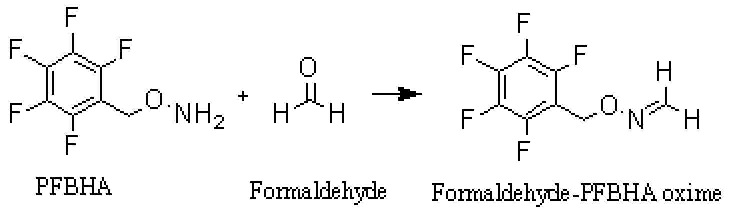

(A) Formaldehyde

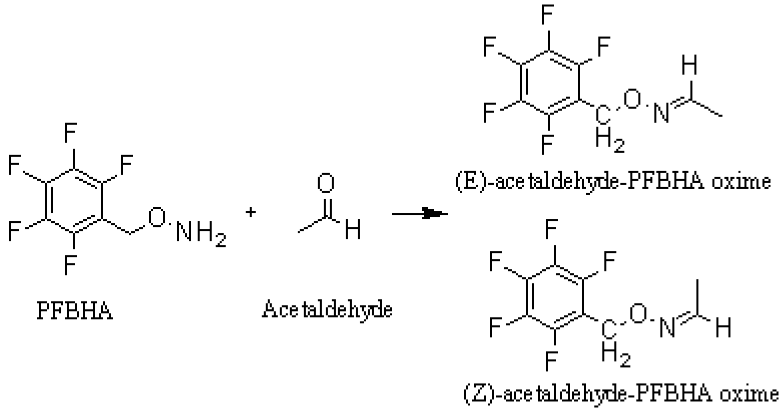

(B) Acetaldehyde

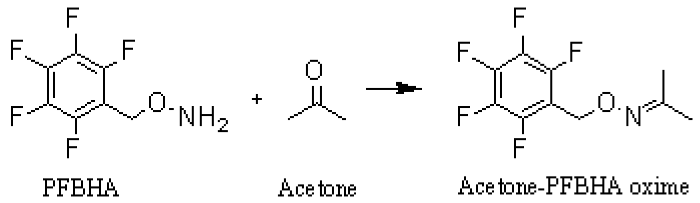

(C) Acetone

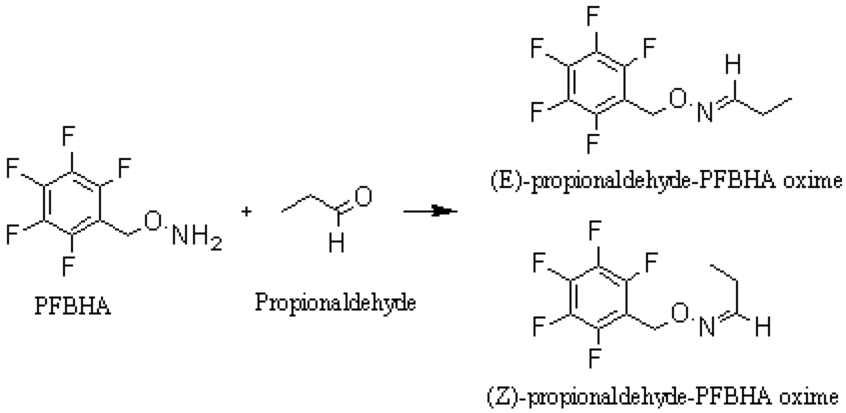

(D) Propionaldehyde

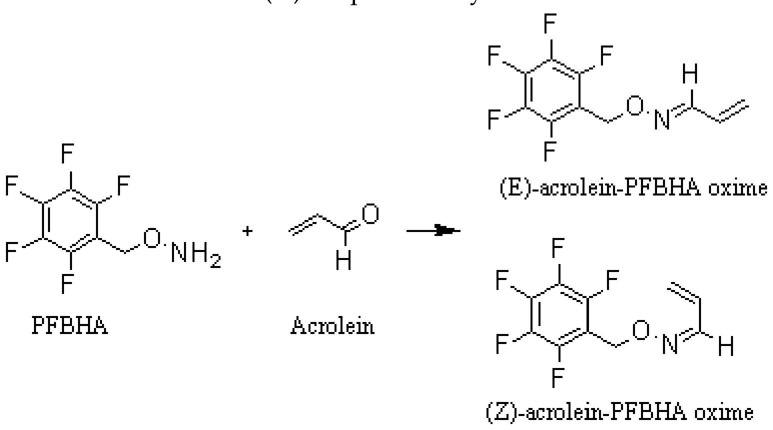

(E) Acrolein

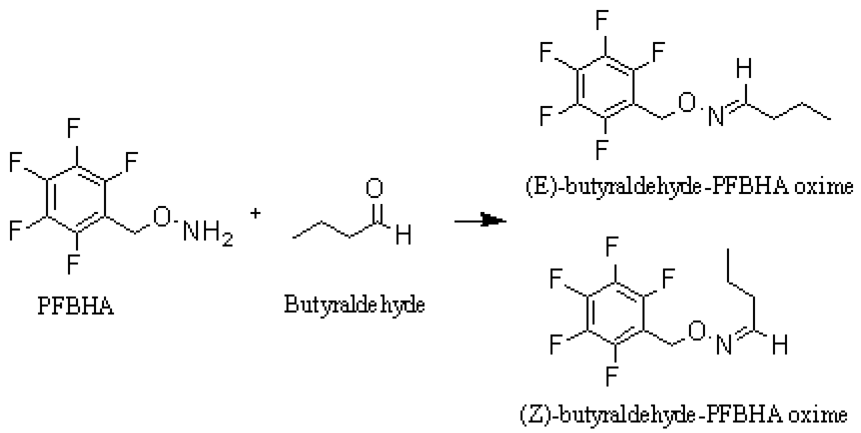

(F) Butyraldehyde

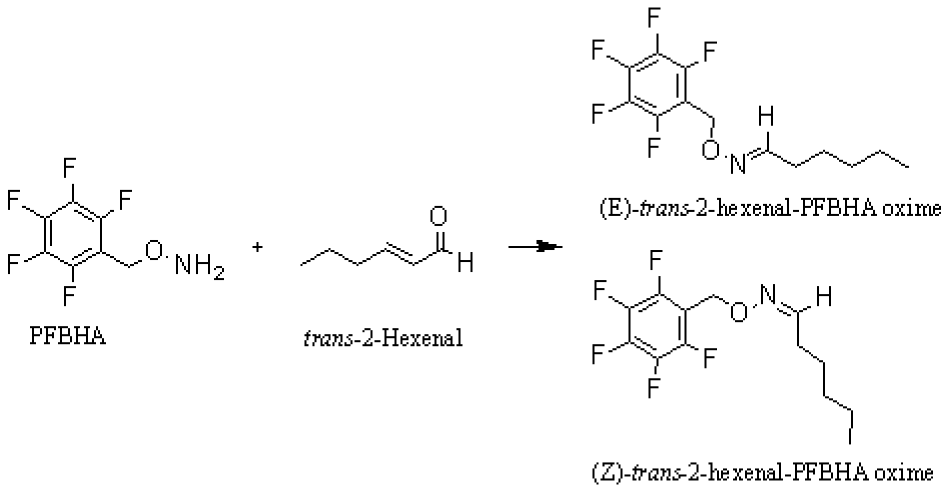

(G) *trans*-2-Hexenal

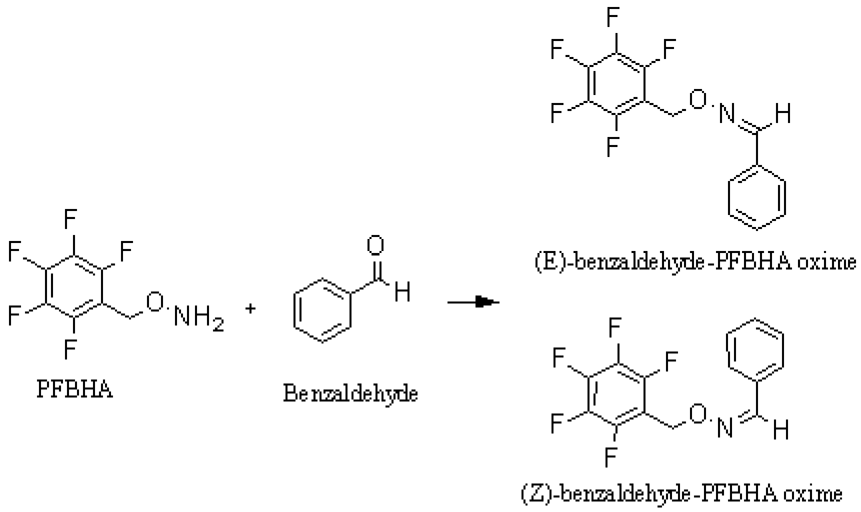

(H) Benzaldehyde

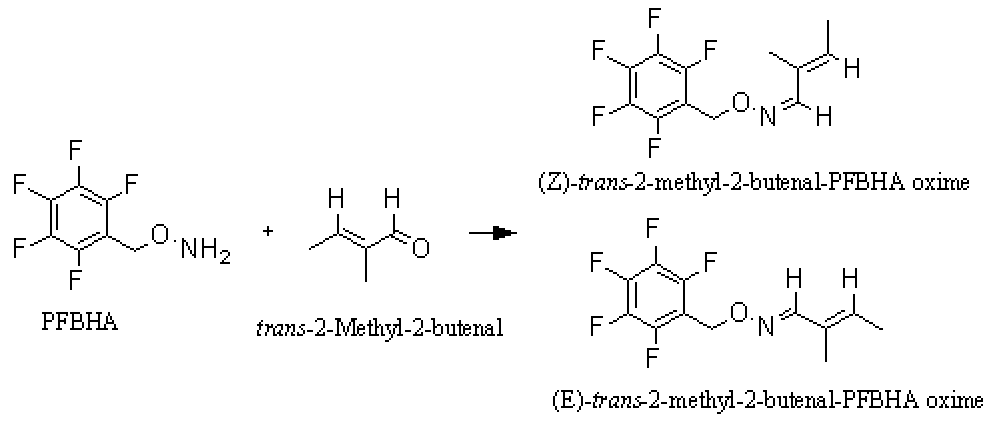

(I) *trans*-2-Methyl-2-butenal

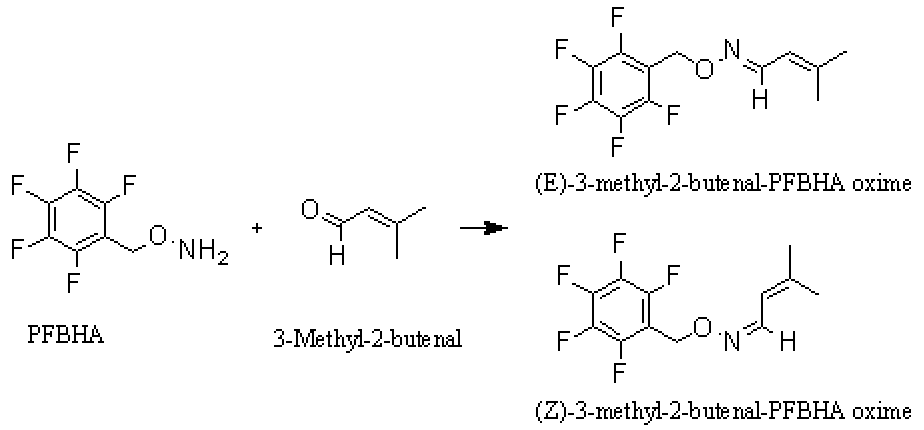

(J) 3-Methyl-2-butenal

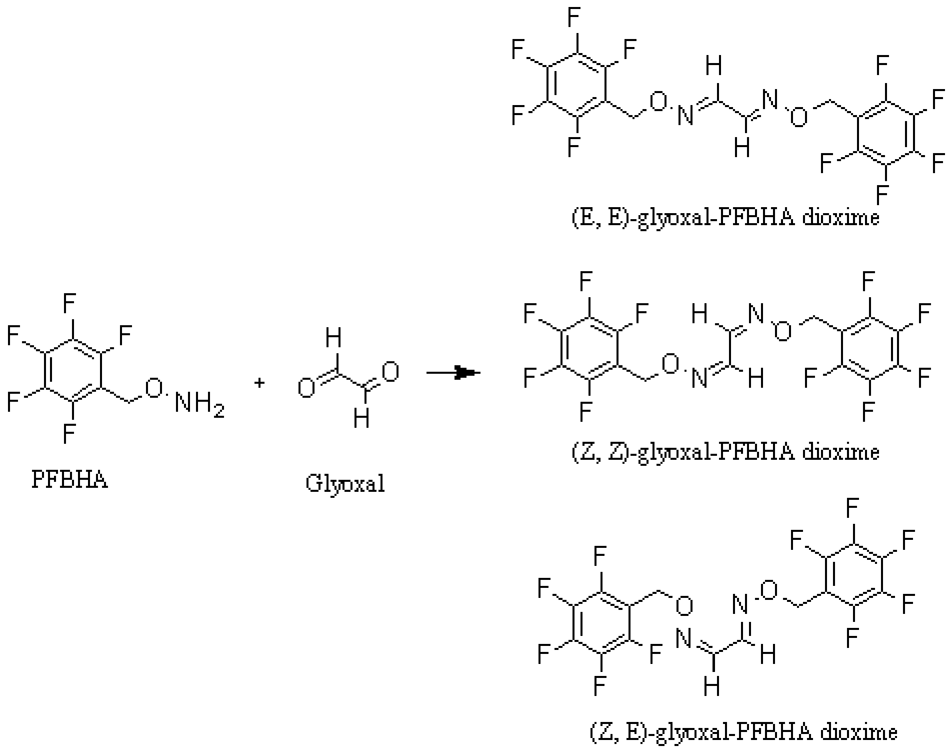

(K) Glyoxal

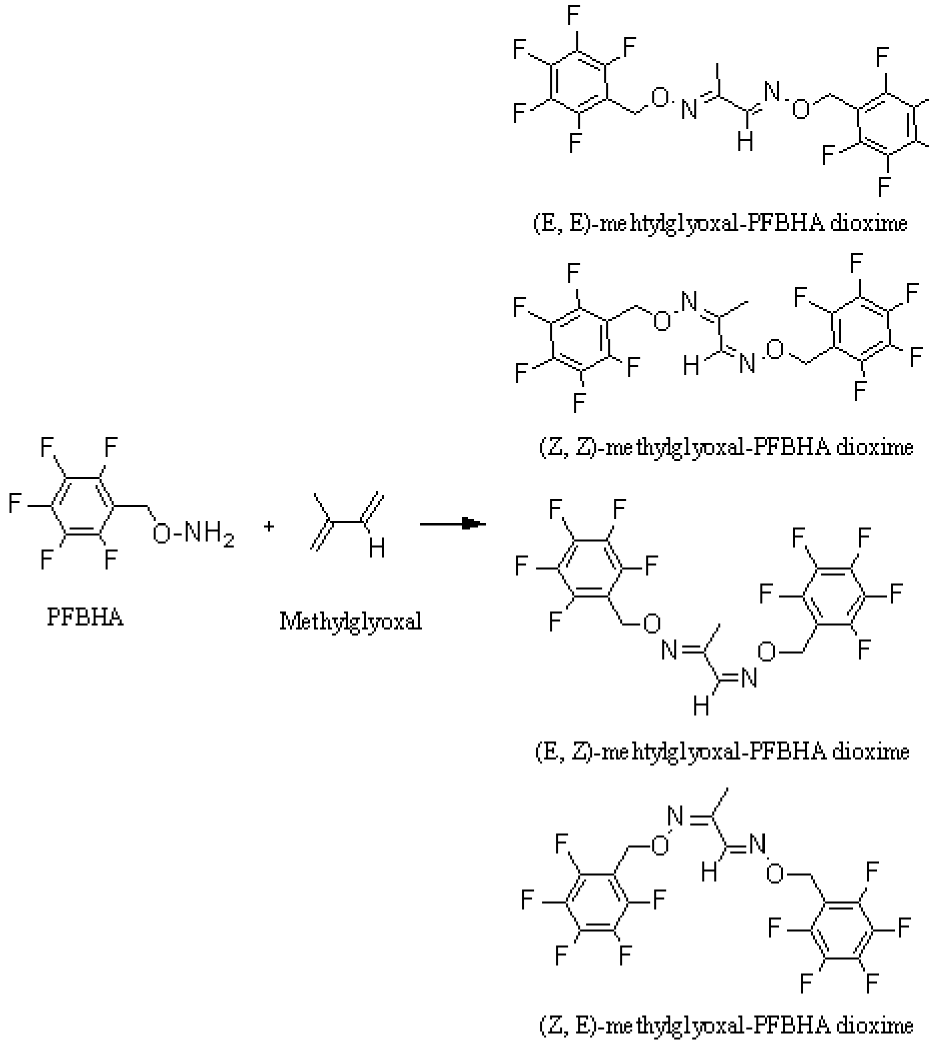

(L) Methylglyoxal

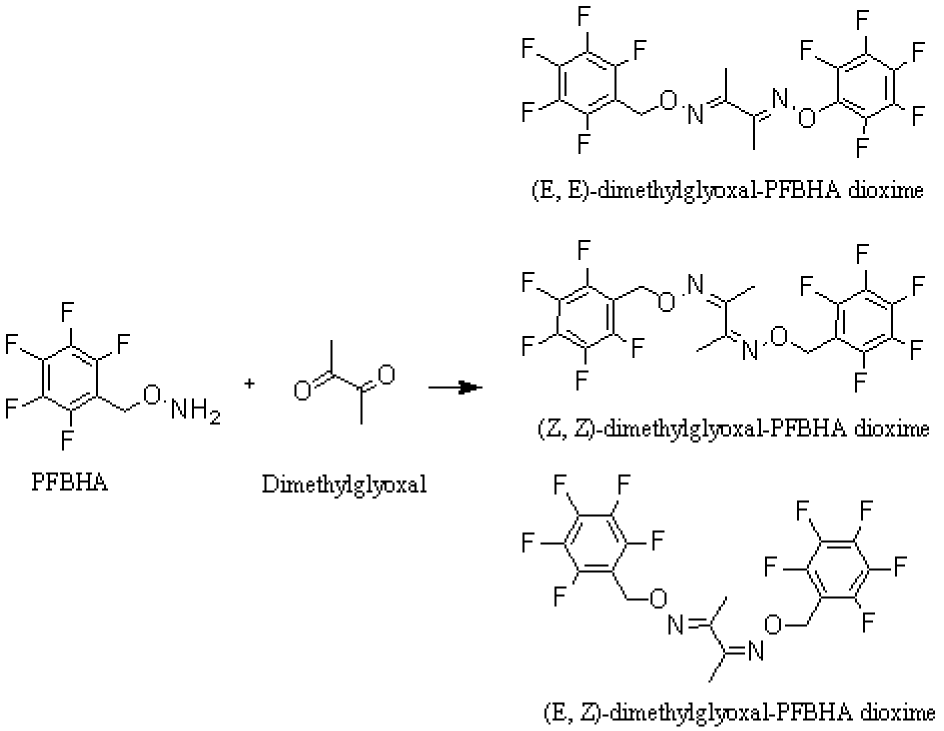

(M) Dimethylglyoxal

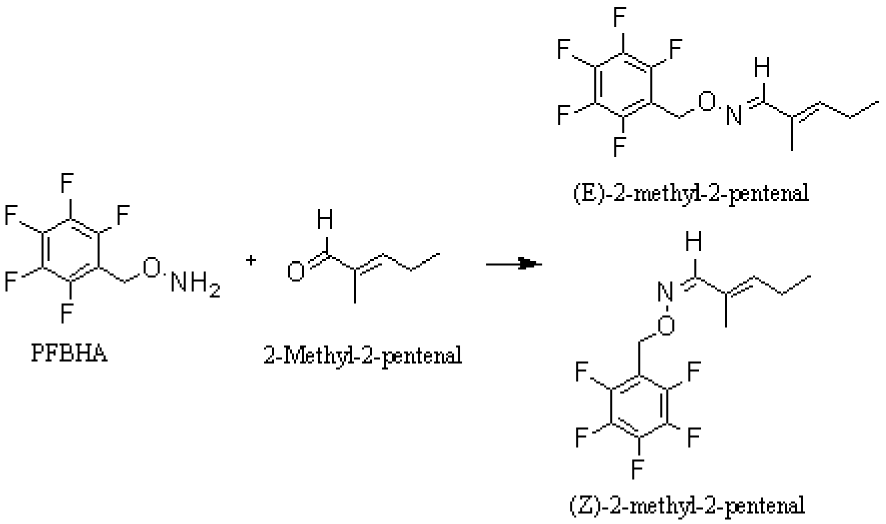

(N) 2-methyl-2-pentenal

Scheme S1. Derivatization between carbonyls (identified in Table 1) and PFBHA, and their oxime derivatives for **(A)** formaldehyde, **(B)** acetaldehyde, **(C)** acetone, **(D)** propionaldehyde, **(E)** acrolein, **(F)** butyraldehyde, **(G)** *trans*-2-hexenal, **(H)** benzaldehyde, **(I)** *trans*-2-methyl-2-butenal, **(J)** 3-methyl-2-butenal, **(K)** glyoxal, **(L)** methylglyoxal, **(M)** dimethyglyoxal, and **(N)** 2-methyl-2-pentenal.

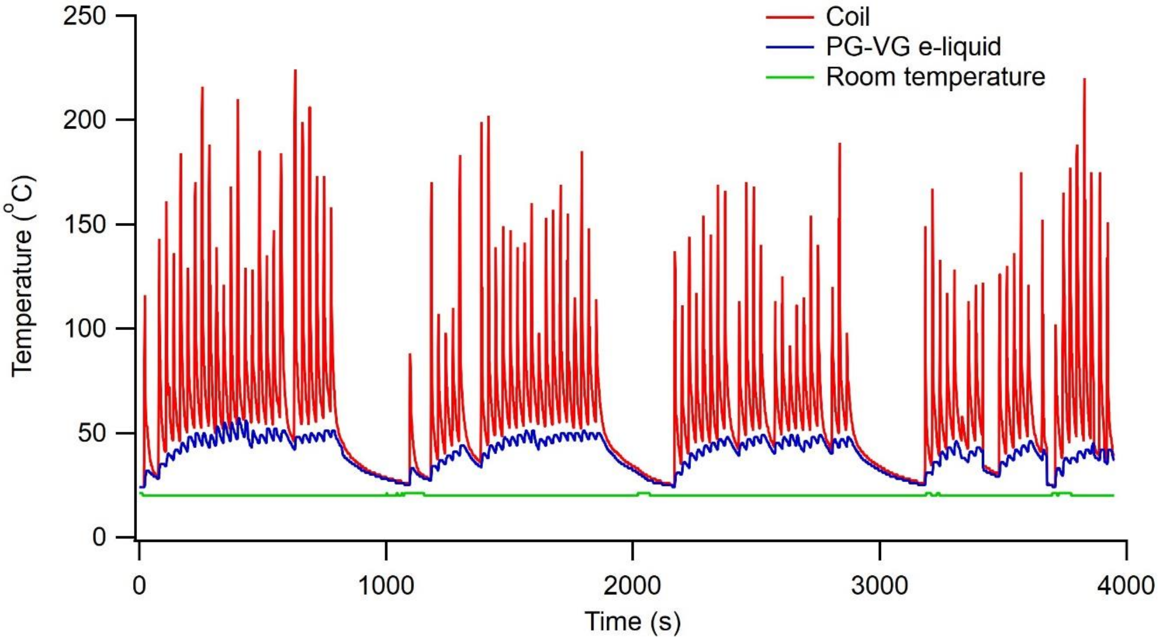

Figure S1. Temperature profile of the coil (red), PG-VG e-liquid (blue), and room temperature (green) measured by K-type thermocouple wires.
